# Replicable brain signatures of emotional bias and memory based on diffusion kurtosis imaging of white matter tracts

**DOI:** 10.1002/hbm.24874

**Published:** 2019-11-26

**Authors:** Thomas Welton, Ben E. Indja, Jerome J. Maller, Jonathon P. Fanning, Michael P. Vallely, Stuart M. Grieve

**Affiliations:** ^1^ Sydney Translational Imaging Laboratory, Heart Research Institute The University of Sydney Camperdown New South Wales Australia; ^2^ Sydney Medical School The University of Sydney Camperdown New South Wales Australia; ^3^ GE Healthcare Richmond Victoria Australia; ^4^ Faculty of Medicine The University of Queensland Brisbane New South Wales Australia; ^5^ The Critical Care Research Group, The Prince Charles Hospital Brisbane New South Wales Australia; ^6^ Department of Cardiothoracic Surgery The Northern Beaches Hospital Sydney New South Wales Australia; ^7^ Department of Radiology Royal Prince Alfred Hospital Sydney New South Wales Australia

**Keywords:** brain, cognition, diffusion kurtosis imaging, emotion, magnetic resonance imaging

## Abstract

Diffusion MRI (dMRI) is sensitive to anisotropic diffusion within bundles of nerve axons and can be used to make objective measurements of brain networks. Many brain disorders are now recognised as being caused by network dysfunction or are secondarily associated with changes in networks. There is therefore great potential in using dMRI measures that reflect network integrity as a future clinical tool to help manage these conditions. Here, we used dMRI to identify replicable, robust and objective markers that meaningfully reflect cognitive and emotional performance. Using diffusion kurtosis analysis and a battery of cognitive and emotional tests, we demonstrated strong relationships between white matter structure across networks of anatomically and functionally specific brain regions with both emotional bias and emotional memory performance in a large healthy cohort. When the connectivity of these regions was examined using diffusion tractography, the terminations of the identified tracts overlapped precisely with cortical loci relating to these domains, drawn from an independent spatial meta‐analysis of available functional neuroimaging literature. The association with emotional bias was then replicated using an independently acquired healthy cohort drawn from the Human Connectome Project. These results demonstrate that, even in healthy individuals, white matter dMRI structural features underpin important cognitive and emotional functions. Our robust cross‐correlation and replication supports the potential of structural brain biomarkers from diffusion kurtosis MRI to characterise early neurological changes and risk in individuals with a reduced threshold for cognitive dysfunction, with further testing required to demonstrate clinical utility.

## 1 INTRODUCTION

A wide range of neurological and psychiatric conditions impact brain function, causing measurable cognitive deficits and emotional dysfunction. Understanding and measuring the cause of these changes would help identify at‐risk individuals, facilitate the development of preventative therapies, and enable early intervention. Unfortunately, psychometric testing is insensitive to early changes in cognition or emotional dysfunction that may precede any formal diagnosis. As such, there is a great clinical need for objective measures that reflect the brain changes that drive cognitive or emotional dysfunction. Many brain disorders are now recognised as being caused by (or are secondarily associated with) network dysfunction; hence, there is great interest in using diffusion MRI (dMRI) as a tool to measure these network changes. dMRI is sensitive to anisotropic diffusion within bundles of nerve axons (Beaulieu, 2002) and can be used to make objective measurements of brain networks (Greicius, Supekar, Menon, & Dougherty, 2009; Mori, Crain, Chacko, & van Zijl, 1999). Recent work on this topic shows disruption to brain connectivity networks at multiple scales is related to emotional and cognitive (dys)function; for example, with dMRI (Fornito, Zalesky, & Breakspear, 2015; Gong & He, 2015; Petersen & Sporns, 2015) and, in combination with functional connectivity using fMRI, multimodal imaging markers of cognition (Jiang et al., 2018; Qi et al., 2018; Sui et al., 2018). Despite this potential and many years of development, high resolution forms of dMRI remain a research tool (i.e., high angular resolution diffusion imaging [Descoteaux, 1999] acquisition schemes or other forms of multi‐shell dMRI), with the only widespread, routine, substantive clinical application being limited usage for surgical planning (Fernandez‐Miranda et al., 2008). The goal of this study was to use high angular resolution dMRI to create a replicable, robust and objective marker of brain changes relating directly to cognitive and emotional functions.

Despite a considerable volume of research, there is still no meaningful clinical usage of multi‐shell dMRI data, such as is collected as part of large‐scale initiatives such as Alzheimer's Disease Neuroimaging Initiative and the Human Connectome Project (HCP). In the clinic, the effects of acute or chronic brain injuries are measured by radiologists using “conventional” MRI techniques such as proton‐density, T2‐weighted and T1‐weighted imaging, with observations usually limited to qualitative grading of imaging features (i.e., normal, mild, moderate, or severe). dMRI is part of this routine clinical imaging battery but is restricted to diffusion weighted imaging (DWI), usually calculated as the trace from an axial low angular resolution single shell dMRI dataset. Probably the most valuable clinical example of “conventional” MRI is the routine use of DWI to detect ischaemic infarction, where lesions with restricted diffusion (low ADC value) correspond quite precisely to a pathologically accurate diagnosis of focal infarction. While this is an extremely important technique, it makes no use of the higher order information available in a dMRI dataset and is only really useful for detecting a stroke for a period of 2–3 weeks following the infarction.

Many brain injuries do not confer observable signs or symptoms by conventional clinical means. Even when imaging is performed, this is often using computed tomography (CT) or conventional MRI. Such conditions are most often reported as “normal,” contrasting with the emerging consensus view that there is often (non‐visualised) ultrastructural damage in many cases. Indeed, the percentage of abnormal CT scans following head injury is approximately 10% (Pandor et al., 2011), despite post‐injury cognitive or emotional dysfunction in these cases occurring chronically at reported rates between 25 and 65% (Dischinger, Ryb, Kufera, & Auman, 2009; Kreutzer, Seel, & Gourley, 2001; Seel et al., 2003). Even in the presence of visible changes such as small embolic foci of restricted diffusion following surgical cases, the DWI change does not accurately predict these outcomes, likely because the extent of underlying damage is not strongly correlated to these “positive” imaging signs (Indja, Woldendorp, Vallely, & Grieve, 2019).

An emerging technique being applied to investigate brain structure is diffusion kurtosis imaging (DKI). DKI advances the conventional model of dMRI by accounting for deviations from the normative Gaussian pattern of diffusion (Steven, Zhuo, & Melhem, 2013; Wu & Cheung, 2010). DKI‐based metrics are therefore independent from diffusion tensor‐based measures and reflect heterogeneity of the tissue. DKI has been shown to improve the sensitivity and specificity of diffusion measurements in a range of diseases, including concussion (Lancaster et al., 2016), motor neurone disease (Welton et al., 2019) and depression (Kamiya et al., 2018). The improved ability of DKI to detect variation in brain diffusion characteristics may enable new effective imaging markers.

Here, we test the spatial covariance of cognitive and emotional domains with brain structure in a large healthy population using DKI. Using these data, we sought to form robust signatures of the structures that underpin normal cognitive and emotional function. We reasoned that such signatures may prove to be useful as a tool to identify patients with an increased vulnerability to neurological injury, early disease processes, or potentially to measure functionally meaningful changes secondary to sub‐clinical brain injury. We tested the replicability and consistency of these structural brain signatures in independent datasets since any potential clinical application mandates robust and repeatable metrics.

## 2 MATERIALS AND METHODS

### 2.1 Participants

To test the robustness of our findings, we developed our analyses in a “Discovery Cohort” and attempted to replicate the key outcomes in a “Replication Cohort.” Our Discovery Cohort comprised 203 healthy individuals drawn from the Chronic Diseases Connectome Project (CDCP). Participants were free from psychiatric or neurological diagnoses, and we included all ages, genders and levels of education in the study. Written informed consent was obtained, and the study had institutional ethics board approval (Macquarie Medical Imaging; 5201500943). The Replication Cohort comprised 1,064 healthy subjects from the HCP (Young Adult Cohort; [Van Essen et al., 2013]).

### 2.2 Cognitive and emotional testing

Neurocognitive testing was performed using the computer‐based WebNeuro battery (Brain Resource Inc., Sydney, New South Wales, Australia; Silverstein et al., 2007). This validated battery of tests reports on 15 cognitive and emotional sub‐scores derived from multiple tasks: negativity bias, emotional resilience, social skills, depression, anxiety, stress, motor tapping, impulsivity, attention, information processing, memory recognition, executive function, verbal interference, emotional identification and emotional bias.

### 2.3 Magnetic resonance imaging

MRI was performed using a 3‐Tesla GE Discovery MR750w MRI scanner (General Electric Healthcare, Milwaukee, Wisconsin) at Macquarie Medical Imaging, Macquarie University Hospital (Sydney, New South Wales, Australia) using a 32‐channel Nova head coil (Nova Medical, Wilmington, Massachusetts). A contiguous AC‐PC aligned sagittal MPRAGE PROMO T1‐weighted image was acquired with the following parameters: TR = 8.39 ms, TE = 3.17 ms, TI = 900 ms, flip angle = 8°, matrix = 256 × 256, 198 slices, voxel dimensions = 1 mm isotropic. dMRI data were acquired with a multi‐shell multi‐band blipped CAIPI (Setsompop et al., 2012) sequence with a phase offset applied to each multi‐band component and a reversed phase‐encode correction. The parameters were as follows: 140 unique gradient directions (25 volumes at *b* = 700, 40 volumes at *b* = 1,000, 75 volumes at *b* = 2,800) and eight interleaved *b* = 0 volumes, TR = 3,245 ms, TE = 100 ms, flip angle = 90°, matrix = 128 × 128, 66 slices, 2 mm isotropic voxels, FOV = 240 mm, multi‐band factor = 3.

### 2.4 Image processing

First, non‐brain tissues were removed from the images using BET (Smith, 2002). TOPUP was used to correct susceptibility‐induced off‐resonance field artefacts using the reverse phase‐encoded images (Andersson, Skare, & Ashburner, 2003). Artefacts from eddy currents and subject head motion were removed (Andersson & Sotiropoulos, 2016). Then, diffusion and diffusion kurtosis parametric maps were created from tensors estimated using Diffusion Kurtosis Estimator (Tabesh, Jensen, Ardekani, & Helpern, 2011). These parameters were: axial diffusivity (DAxial), radial diffusivity (DRadial), fractional anisotropy (FA), axial kurtosis (KAxial), radial kurtosis (KRadial), kurtosis FA (KFA), mean diffusivity (MD) and mean kurtosis (MK).

Fractional anisotropy data were then aligned to a common space (the MNI152 average brain Mazziotta, Toga, Evans, Fox, & Lancaster, 1995) using the nonlinear registration tool, FNIRT (Andersson, Jenkinson, & Smith, 2007). We performed tract‐based analysis of diffusion kurtosis scalars and their covariance with each cognitive component using tract‐based spatial statistics (TBSS; Smith et al., 2006). In short, a mean FA image was created and thresholded using default settings (0.2) to create a mean FA “skeleton,” which represents the centres of all tracts common to the group. Each subject's aligned diffusion and kurtosis data were projected onto this skeleton and the resulting data fed into voxelwise cross‐subject statistics.

T1‐weighted images were analysed using voxel‐based morphometry (VBM; Douaud et al., 2007). The grey matter was segmented from these images using FAST (Andersson et al., 2007) and, as above, non‐brain tissues were removed before registering the images to the MNI brain. A study‐specific, symmetric template was created by averaging these images and flipping them along the *x*‐axis. Then, all grey matter images were nonlinearly registered to the template and corrected for local expansion or contraction from the nonlinear component of the registration. The corrected grey matter images were then smoothed with an isotropic Gaussian kernel with a sigma of 3 mm.

### 2.5 Statistics

The significance threshold for all tests was set at 0.05. We initially excluded subjects who were outliers in any cognitive test (±10 SDs relative to the WebNeuro normative cohort [*n* > 10,000], Silverstein et al., 2007) or who had missing or incomplete cognitive or imaging data.

Raw neurocognitive tests scores were transformed into *Z*‐scores. Where multiple measures were reported for a single test (i.e., the continuous performance test consisted of response time plus number of errors) a composite score was calculated, as was the case where multiple tests measured a single neurocognitive domain, based on previous work by Goodkind et al. (2015). This resulted in 15 individual scores which underwent further data reduction using principal component analysis with a varimax rotation. One‐sample *t*‐tests employing the WebNeuro normative cohort were used to test whether subjects were cognitively normal.

For TBSS and VBM analyses, a voxelwise general linear model was applied, using permutation‐based non‐parametric testing in randomise/PALM (Winkler, Ridgway, Webster, Smith, & Nichols, 2014). Across space, the family‐wise error rate was controlled, and threshold‐free cluster enhancement applied to account for multiple comparisons. The family‐wise error rate was also controlled across contrasts (Winkler et al., 2016).

### 2.6 Construction and validation of structural brain signatures

Based on the results of the above analyses, we used the Discovery Cohort to combine the best identified features to form potential “structural brain signatures” sensitive to functional measures. To define the signatures, we combined the significant findings from the tract‐based analyses of the emotional bias and emotional memory components: at the local peak coordinates of the clusters which were strongly significant (*p* < .01), we extracted the mean value in each cluster and multiplied them together. Linear regression of this score against the cognitive and emotional measures was used to test sensitivity.

A “sham signature” was also created from the left corticospinal tract using the same methods, an anatomically proximal white matter tract with a low probability of structure–function correlation. This was used to test specificity (anatomically and pertaining to the cognitive components). A mask was placed along the corticospinal tract from the level of the cerebral peduncles to the precentral gyrus white matter and transformed to the individual subjects' space. These voxels were identified from probabilistic diffusion tractography performed in a single high resolution diffusion dataset gathered using a head‐only MRI scanner with high‐performance gradients (Foo et al., 2018) and registered to the individual subjects' brain images (Callaghan et al., 2018; Maller et al., 2019). To form the sham signatures, the relevant mean diffusion metrics were extracted from these regions and multiplied together, as described above (Supporting Information S1).

### 2.7 Replication analysis

Emotional bias and recognition were assessed in the Replication Cohort using the Penn Emotion Recognition Test (ER40; number of correct responses; Gur et al., 2001), which we interpreted as an analogue to our emotional bias and emotional memory components. The same initial processing steps as in the Discovery Cohort were followed (estimation of diffusion and kurtosis scalars, removal of non‐brain tissues, registration to a standard space) but using the TBSS skeleton from the Discovery Cohort for consistency. Then, the structural brain signatures identified in the Discovery Cohort were applied to obtain a score for each signature and linear regression was performed to test whether the same finding could be replicated.

## 3 RESULTS

### 3.1 Demographics and cognition

**Figure 1:**
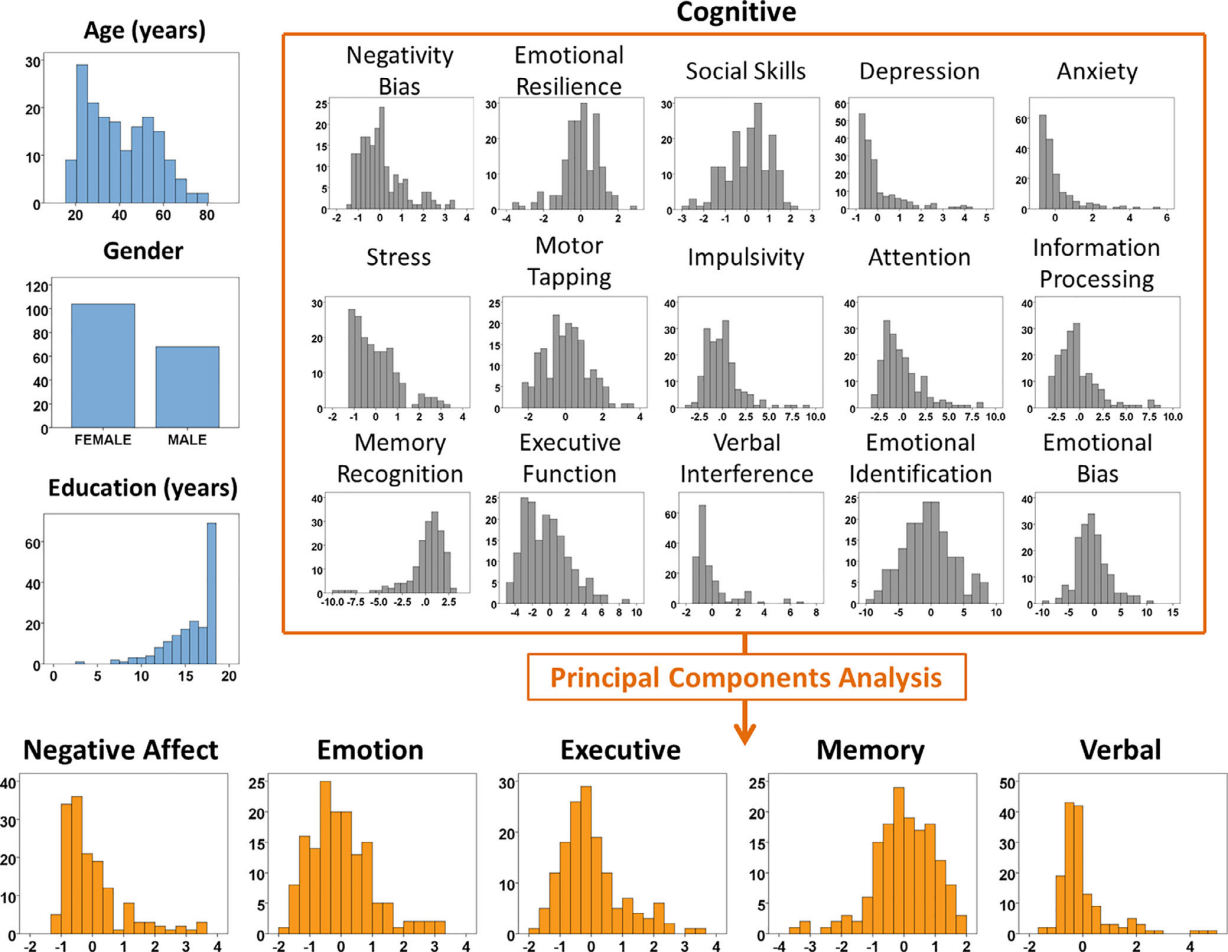
Histograms for each of the demographic (blue) and cognitive variables used in the study, and the results of principal components analysis (orange)

Characteristics of the Discovery Cohort are shown in Table 1. After the removal of extreme outliers in cognition (*n* = 20) and subjects with incomplete data (*n* = 11), 172 subjects remained for full analysis. There was a wide spread of ages centred on 40 years (median; 27–53 interquartile range) and a slight female gender bias (60%; Figure 1). Our sample was considered normal for cognition and emotion, as demonstrated by the distributions of age‐ gender‐ and education‐corrected scores (one‐sample *t*‐tests: *p* > .05; Supporting Information S2).

**Table 1:** Demographic and cognitive characteristics of the discovery cohort (*n* = 172)

	Count	Mean	*SD*	Median	Interquartile range
*Demographic*					
*N*	172				
Gender, % female	60.50				
Age, years		40.34	14.97	39.00	26.00
Education, years		15.70	2.89	17.00	4.00
*Cognitive*					
Negativity bias		0.01	1.02	−0.13	1.27
Emotional resilience		0.00	1.00	0.05	1.26
Social skills		0.03	1.00	0.11	1.43
Depressed mood		0.00	1.00	−0.44	0.82
Anxiety		0.03	1.02	−0.31	0.72
Stress		0.02	1.01	−0.23	1.46
Motor tapping		0.02	1.15	0.01	1.58
Impulsivity		−0.06	1.86	−0.33	2.06
Attention		−0.09	2.13	−0.59	2.47
Information processing		−0.25	2.16	−0.66	2.20
Memory		0.13	2.13	0.54	1.88
Executive		−0.41	2.55	−0.63	3.50
Verbal interference		−0.11	1.42	−0.62	0.93
Emotional identification		−0.39	3.84	−0.60	5.10
Emotional bias		−0.40	3.12	−0.85	3.62

*Note*: Cognitive data are from the WebNeuro computerised test battery and are unadjusted for any demographic variable. Some measures are expressed as *Z*‐scores, while others are composites, thus the centre and spread are often 0 and 1, respectively. All cognitive data are arranged such that a positive score indicates better performance.

The cognitive and emotional data were used to derive five principal components which, together, explained 66% of total variance. Based on their component loadings (Supporting Information S3), we named these “negative affect,” “emotional bias,” “executive,” “emotional memory” and “verbal,” and used them as the independent variables for the following spatial analyses of brain structure.

The negative affect component was positively weighted toward greater stress, negative emotional bias, depression and anxiety, and negatively weighted for emotional resilience and social skills. The emotional bias component was non‐specific and was weighted toward emotional recognition and bias. The executive component was equally weighted for executive performance in a maze task and attentional switching, and negatively weighted for memory. The emotional memory component was weighted toward both memory and emotional resilience and might therefore be specific to emotional memory. Lastly, the verbal component was primarily weighted toward performance in a verbal interference test. The negative affect component was inverted so that higher values reflected better performance.

### 3.2 Voxelwise analysis of diffusion kurtosis scalars

**Figure 2:**
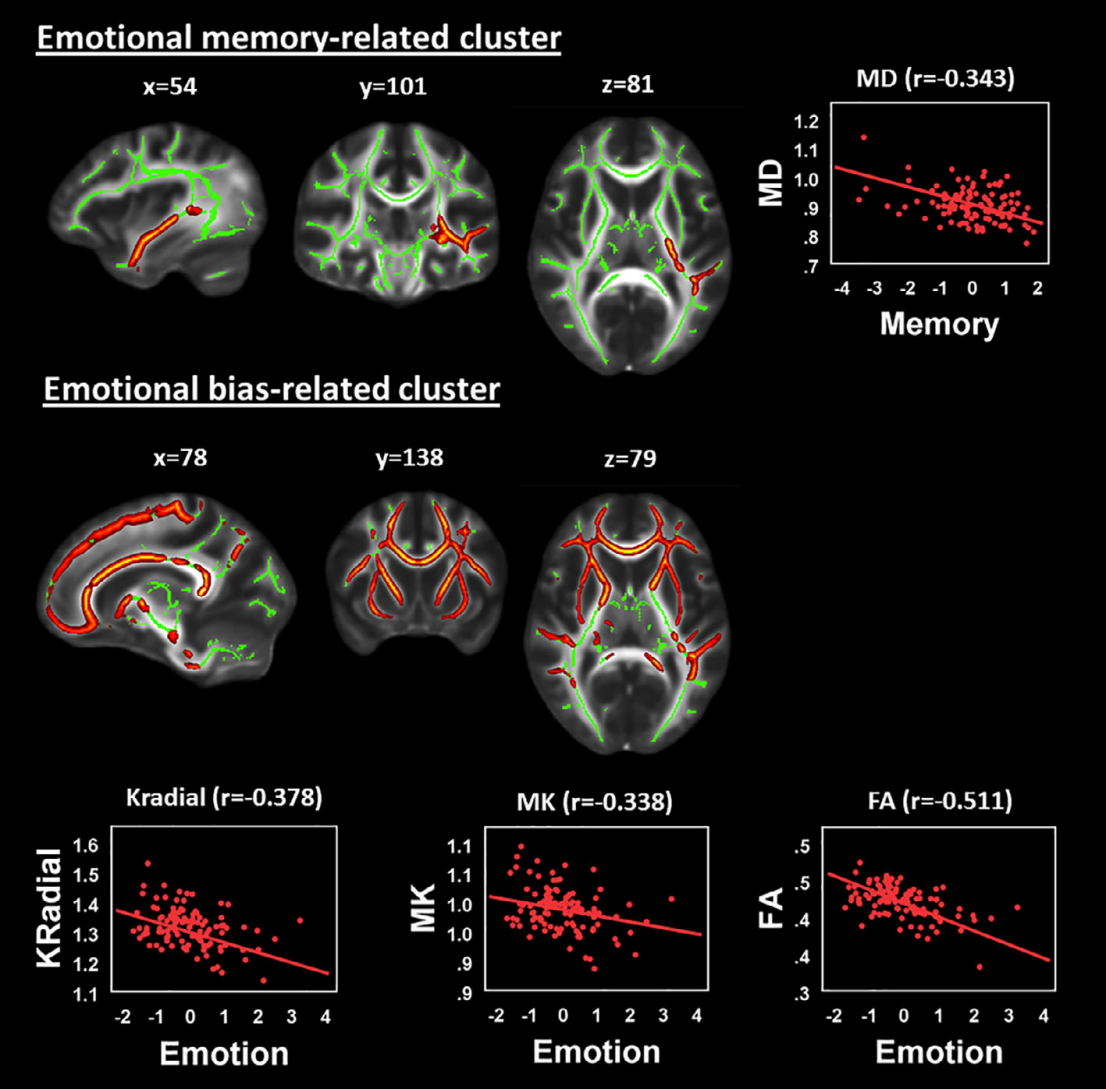
Results of the tract‐based spatial statistics analysis using diffusion kurtosis scalars. Significant clusters (in red–orange) have been enlarged for clarity. The FA skeleton is shown in green, overlaid on the MNI average FA image. Scatter plots are only shown for metrics with clusters having *p* < .01. FA, fractional anisotropy

In the tract‐based analysis of diffusion kurtosis scalars and their covariance with each of the five cognitive‐emotional components, we found two main significant clusters across multiple diffusion and kurtosis scalars for two components: emotional bias and emotional memory (Table 2; Figure 2).

**Table 2:** Tests with significant findings and the direction of the effect for the tract‐based and whole‐brain analyses

Component	DAxial	DRadial	MD	FA	KAxial	KRadial	MK	KFA
*Tract‐based*								
Negative affect								
Emotional bias		**+**	**+**	**−**	**−**	**−**	**−**	**−**
Executive								
Emotional memory	**−**	**−**	**−**					
Verbal								
*Whole‐brain*								
Negative affect	**+**	**+**	**+**					
Emotional bias		**+**	**+**	**−**	**−**	**−**	**−**	**−**
Executive								
Emotional memory	**−**	**−**	**−**					**+**
Verbal								

*Note*: Significance was determined by a voxelwise *p*‐value <.05 (corrected for the family‐wise error rate within clusters formed using a threshold‐free approach [Smith & Nichols, 2009]). “+” indicates a direct relationship, that is, a higher diffusion metric relating to a higher component score and, vice‐versa, “**−**” indicates an inverse relationship.

Abbreviations: FA, fractional anisotropy; KFH, kurtosis FA; MD, mean diffusivity; MK, mean kurtosis.

A significant emotional memory‐related cluster was identified that extended along the entire left medial temporal lobe and left posterior limb of the internal capsule at the level of the thalamus (upper row, Figure 2, Table 3). This cluster was significant for the DAxial, DRadial and MD scalars, varying from 1.4 to 3.4 ml in volume across diffusion scalars (Table 3), representing an inverse relationship.

**Table 3:** Information for significant clusters identified in the tract‐based spatial statistics and whole‐brain spatial statistics analyses

Cluster name	Diffusion/kurtosis scalar	Extent (ml)	Peak coordinate (MNI‐space)	*t* (peak)	*t* (mean)	*p* (FWE‐corrected)
*Tract‐based diffusion/kurtosis*						
Left medial temporal—emotional memory						
	DAxial	1.38	22, 51, 36	−4.56	−2.57	.03
	DRadial	1.59	27, 51, 35	−4.87	−3.05	.02
	MD	3.36	27, 56, 31	−5.29	−2.79	<.01
Frontal—emotional bias						
	DRadial	28.06	38, 65, 52	4.79	1.89	.02
	MD	4.45	38, 66, 52	3.67	2.22	.04
	FA	44.96	25, 78, 41	−5.01	−1.70	<.01
	KAxial	3.68	62, 78, 44	−4.68	−2.35	.02
	KRadial	73.695	63, 76, 45	−5.22	−1.86	<.01
	MK	71.15	59, 75, 45	−5.31	−2.03	<.01
	KFA	1.78	62, 77, 45	−4.16	−2.21	.02
*Whole‐brain diffusion/kurtosis*						
Thalamus/fimbria—Negative affect						
	DAxial	0.19	52, 51, 41	5.42	4.83	.04
	DRadial	2.20	52, 51, 41	5.60	4.15	.04
	MD	1.11	52, 51, 41	5.50	4.47	.04
*Whole brain* (*grey matter volume*)						
None						

Abbreviations: FA, fractional anisotropy; KFH, kurtosis FA; MD, mean diffusivity.

A single large cluster was associated with emotional bias and, while it varied in extent across scalars (1.8 ml in KFA to 73.7 ml for KRadial; Table 3), the cluster was primarily distributed across the frontal lobe, extending into the parietal and temporal lobes. At *p* < .05, this cluster was significant in all diffusion and kurtosis scalars except DAxial and, at *p* < .01, only in the MK (*r* = −.34), KRadial (*r* = −.38) and FA (*r* = −.51) scalars.

No significant tract‐wise clusters were identified for the negative affect, executive or verbal components. Inspection of the unthresholded distributions of *t*‐statistic maps for these components revealed two non‐significant trends (*t* > 2.5). First, the DAxial, DRadial, FA, KRadial, MD, MK and KFA scalars covaried with the negative affect component in the thalami and posterior cingulate (Supporting Information S4). Second, the MD, MK and KAxial scalars covaried directly with the verbal component in the anterior commissure.

As a confirmatory test, we performed a similar analysis including all intracranial voxels (again, using threshold‐free cluster enhancement and *p* < .05). In addition to the clusters identified in the tract‐based analysis described above, we found one cluster reflecting grey matter volume. This cluster involved the right hippocampus fimbria and right thalamus, exhibiting direct covariance with the negative affect component in the DAxial, DRadial and MD scalars (Table 2, Supporting Information S5).

The location of the identified emotional memory cluster was consistent with the known role of the medial temporal lobe in both emotion and memory function, and that of sensory fibres derived from the thalamus and its interconnectivity with the hippocampus (Maller et al., 2019). Similarly, the specific role of the frontal lobe in emotional bias and identification is well established (Phillips, Drevets, Rauch, & Lane, 2003). Therefore, we sought to test the functional relevance of the identified clusters against known functional hubs for the respective cognitive and emotional functions.

**Figure 3:**
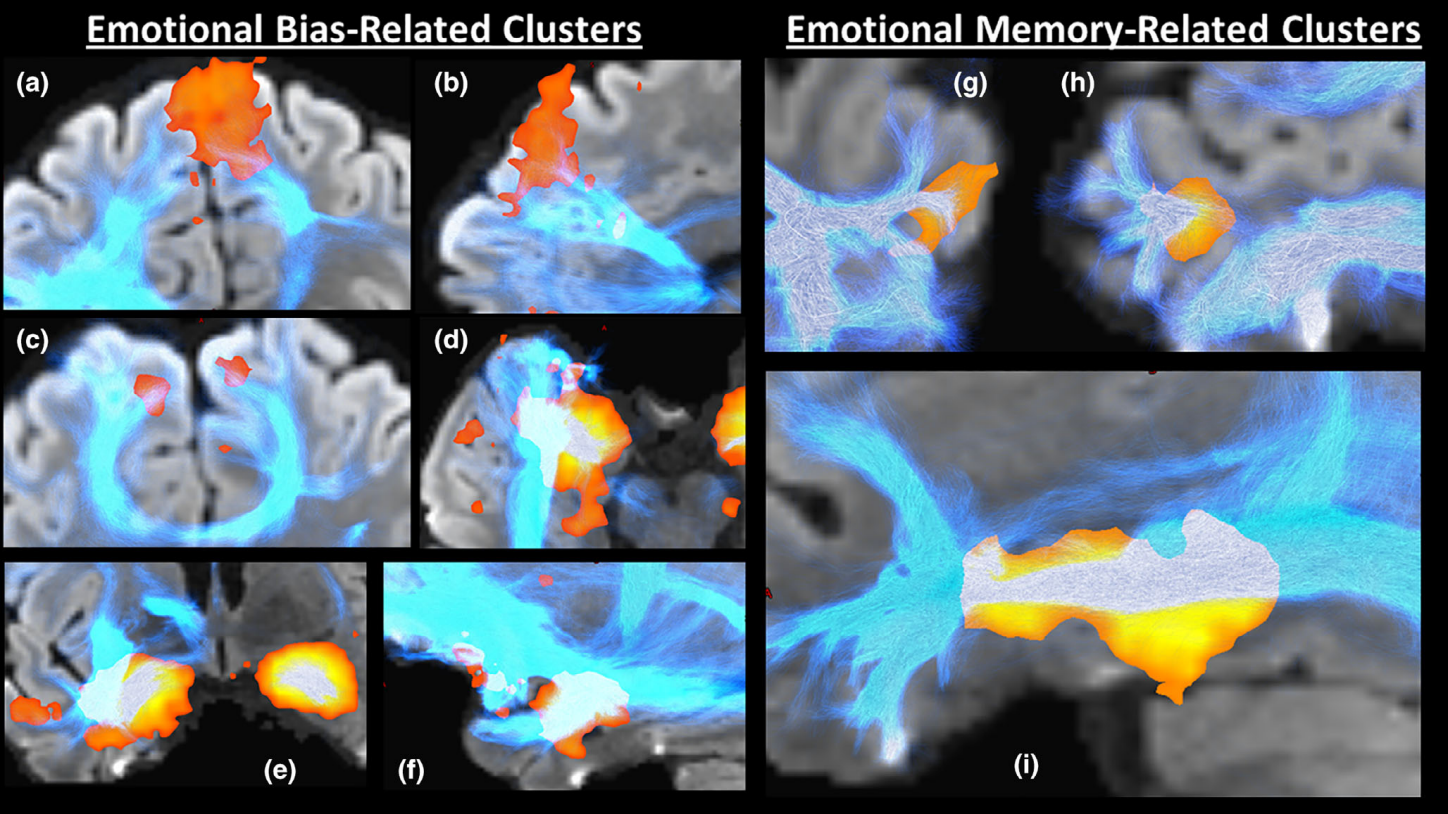
Probabilistic diffusion tractography of the identified tracts. In blue are the probabilistic streamlines generated using the significant clusters as a seed region. The orange regions are gathered from automated NeuroSynth meta‐analyses of functional MRI studies of emotional memory or emotional bias. The subfigures are as follows: (a, b, c) medial prefrontal cortex emotional bias‐related cluster with intersecting tracks bilaterally mostly via the corpus callosum superior genu in order of coronal, sagittal and axial views, (d, e, f) hippocampal emotional bias clusters intersected in the right hemisphere by tracks primarily from the uncinate and inferior longitudinal fasciculi in order of axial, coronal and sagittal views, (g, h) left middle temporal gyrus emotional memory cluster intersected by tracks from the inferior longitudinal fasciculus from axial and sagittal views, and (i) left hippocampus‐amygdala emotional memory cluster intersected by inferior longitudinal fasciculus tracks in a sagittal view

To do this, we used each of the significant clusters to seed probabilistic diffusion tractography on a single ultra‐high angular resolution diffusion dataset, gathered using a head‐only MRI scanner with high‐performance gradients (Supporting Information S6). We visualised the result alongside automated meta‐analytic topic maps gathered from NeuroSynth, to show grey matter regions associated with emotion and memory (https://neurosynth.org; [Yarkoni, Poldrack, Nichols, Van Essen, & Wager, 2011]; emotional memory topic number 272 [501 studies]; emotional bias topic number 20 [669 studies]; version 5, July 2018). Qualitatively, the visualised tracts had good correspondence with these identified grey matter regions, attesting to the functional relevance of our clusters (Figure 3).

### 3.3 Voxel‐based morphometry

We detected no clusters of significant voxels representing covariance of the cognitive‐emotional components with grey matter volume. Inspection of the non‐thresholded *t*‐statistic maps revealed a sub‐threshold trend (*t* > 2.5) in the negative affect component for greater grey matter volumes symmetrically in the hippocampi (Supporting Information S7).

### 3.4 Construction and validation of structural brain signatures

The emotional bias signature was normally distributed (Shapiro–Wilk test: W = 0.99, *p* = .55) and uncorrelated with age (*r* = −.14, *p* = .14), gender (*r* = −.06, *p* = .51) and education (*r* = −.13, *p* = .17). Inspection of a scatter plot suggested that the relationship between the signature and the component was approximately linear. Linear regression analysis of the signature against the component scores revealed a moderate but significant inverse relationship (*F*[1,172] = 25.67, *R* ^2^ = −.18, *p* < .001, Supporting Information S8). When adding age, gender and education by backward selection, none were included in the model.

The emotional memory signature was not normally distributed, having a slight positive skew (Shapiro–Wilk test: *W* = 0.97, *p* = .02) and was correlated with age (*r* = .35, *p* < .01) but not with gender (*r* = .002, *p* = .98) or education (*r* = −.05, *p* = .63). Inspection of a scatter plot suggested that the relationship between the signature and the component was approximately linear. Linear regression analysis of the signature against the component scores revealed an inverse relationship (*F*[1,172] = 14.38, *R* ^2^ = −.12, *p* < .001, Supporting Information S8). When age, gender and education were added to the model (by backward stepwise selection), none were included in the final model.

### 3.5 Specificity of the structural brain signatures

To demonstrate selectivity of the two signatures to their respective cognitive components, we repeated the regression analyses using each of the other cognitive components. Regression analyses of the emotional bias signature using the negative affect, executive function, emotional memory and verbal components were all non‐significant (*p* = .26, .21, .92 and .75, respectively; Supporting Information S9). Regression analyses of the emotional memory signature using the negative affect, executive function, emotional bias and verbal components were also all non‐significant (*p* = .23, .22, .30 and .11, respectively; Supporting Information S10). This supports the selectivity of the signatures for their respective emotional domains.

To demonstrate selectivity of the signature to the identified anatomical regions, we repeated the regression analyses using the sham signature. Linear regression of the sham signature against the emotional bias component showed no significant relationship (*F*[1,172] = .10, *R* ^2^ = .03, *p* = .75), illustrating selectivity of the signature to the frontal white matter pathways. Similarly, the emotional memory sham signature regression was not significant (*F*[1,172] = .42, *R* ^2^ = −.01, *p* = .82). Neither age, gender nor education were selected for either model.

### 3.6 Replicability of the structural brain signatures

We then tested whether the same associations between our structural brain signature and emotional bias were replicable in a second, independent, large, healthy Replication Cohort. This cohort had a median age of 29 years (6‐year interquartile range) and a slight female bias (54% female). The ER40 correct responses score (reflecting emotional bias) distribution was negatively skewed (skewness: −0.88; Shapiro–Wilk test: *W* = 0.94, *p* < .01), with a median of 36.0 and an interquartile range of 3.0. Linear regression of the emotional bias signature against the ER40 score, age, gender and education revealed a modest but significant direct effect in the same direction as was observed in the Discovery Cohort (*F*[3,1,061] = 27.28, *R* ^2^ = .07, *p* < .001; age, gender, education not selected). Residuals were approximately normally distributed. We did not test for replicability of the emotional memory component because there were no comparable cognitive data (i.e., testing emotional resilience) available for the Replication Cohort.

## 4 DISCUSSION

The clinical applications of MRI are predominantly limited to the diagnosis of acute injuries or gross physical abnormalities. Even in cases where “changes” are detectable using conventional MRI, these data are not easily quantifiable or predictive of progression to formal cognitive decline or mood disorder. New MRI acquisition and analysis techniques can measure brain networks, so have some potential to change this. In particular, dMRI is the focus of much research, including the HCP which has, to date, generated more than 340 papers from members of the HCP consortium alone (Van Essen & Glasser, 2016). Despite this activity, there is currently no clinically meaningful use of high resolution dMRI. In this article, we aimed to develop objective, replicable dMRI measurements of brain tissue diffusion that predict brain function and that might therefore form candidate markers for objective measurements of brain integrity with further testing in the future.

We applied DKI, a promising dMRI analytical approach sensitive to brain ultrastructure, to map how cognitive function relates to brain structure. Our analysis reflects the most comprehensive and highly powered analysis of this type to date. Our key findings were: (a) the identification of two structural brain signatures which are sensitive and specific to emotional bias and emotional memory; (b) demonstration that these signatures involve white matter pathways, which closely relate to functionally relevant cortical regions linked to these emotional domains; and (c) replication of the primary finding in a second large, independently acquired normal cohort. In identifying associations between microstructural white matter changes and normal variations in neurocognitive function, we show new evidence to support diffusion tensor and kurtosis techniques forming a basis for developing sensitive and specific biomarkers that can readily identify and characterise structurally based brain dysfunction, such as may occur after injury, or in early psychiatric or neurological diseases. Our data were derived from healthy cohorts, and the most realistic potential application is, therefore, as a test to objectively quantify injury and identify subjects who may be more vulnerable to future emotional and cognitive dysfunction. Further work is clearly required to demonstrate utility in the setting of injury or disease.

The identified structural brain signatures were derived from two main clusters, the first of which related to emotional bias and was located throughout the frontal lobe, as well as extending to the parietal and temporal lobes. Emotional dysregulation associated with the frontal lobe (Phillips et al., 2003) is a frequent morbidity observed in many neuropathological states, such as dementia (Goodkind, Gyurak, McCarthy, Miller, & Levenson, 2010), traumatic brain injury (van der Horn, Liemburg, Aleman, Spikman, & van der Naalt, 2016) and surgical brain injury (Indja et al., 2017). Mood disorders and emotional dysfunction are extremely difficult aspects of such conditions to manage and contribute significantly to impaired quality of life. In our data, the emotional bias score was negatively related to the FA and kurtosis‐based metrics, and positively related to the mean and radial diffusivity metrics across the frontal lobe clusters. The direction of this effect suggests that greater performance in our emotional tests (i.e., less bias toward negative emotions) corresponds to greater diffusivity, reduced tissue integrity and reduced cellular complexity in these specific regions (Steven et al., 2013). The neurobiological mechanisms governing these effects may related to the structural integrity of myelin, a theory supported by previous studies of cognitive decline in normal healthy individuals (Madden et al., 2012).

The second cluster was related to emotion and memory and was located along the left medial temporal lobe and left posterior limb of the internal capsule. In contrast, the direction of the effect was different for the emotional memory and MD, axial diffusivity and radial diffusivity relationships: better memory performance corresponding to lower diffusivity consistent with greater density of membranes. The role of the medial temporal lobe and hippocampus in memory (Maller et al., 2019) has been studied extensively; and, in dementia, there is strong evidence of an association with grey matter atrophy (Zakzanis, Graham, & Campbell, 2003). Whilst hippocampal atrophy occurs early, in the case of the medial temporal lobe, grey matter atrophy is often not seen until disease is well established (Zakzanis et al., 2003). The lack of any significant association between grey matter volume and cognition is not surprising given that grey matter volume change is a gross, non‐specific measure which is impacted by many complex factors. Such atrophy is typically regarded as a downstream consequence of neurological and psychiatric dysfunction, whereas white matter changes are hypothesised to be recognisable early in a disease's course (Agosta et al., 2011; Zhuang et al., 2013). This has been demonstrated in the superior ability of the volume of white matter hyperintensities to predict the onset of Alzheimer's disease over hippocampal atrophy (Brickman et al., 2012). Diffusion tensor imaging has also been shown to accurately estimate the presence and timing of traumatic brain injury over traditional imaging techniques (Mac Donald, Dikranian, Bayly, Holtzman, & Brody, 2007).

Following identification and validation of these emotion‐related clusters, we showed that our finding regarding the relationship between emotional bias and diffusion kurtosis characteristics of the matching cluster was replicable in a second, larger cohort. The need for replication of neuroimaging findings has come to the forefront in recent years (Poldrack et al., 2017), spurred by concerns about reliability in psychology research (Simmons, Nelson, & Simonsohn, 2011) and concerns about false positives in fMRI studies (Eklund, Nichols, & Knutsson, 2016). This is exacerbated by the cost of data acquisition and the wide variety and complexity of analyses in the neuroimaging field. In response, practical guidelines for replication in neuroimaging studies have been published (Bakken, 2019; Gorgolewski & Poldrack, 2016), journals have accommodated replication studies (Picciotto, 2018), for example, the *Human Brain Mapping Replication Award* and the creation of a replication category in *NeuroImage*: *Clinical* (Fletcher & Grafton, 2013), and an educational course to teach computation reproducibility has been trialled (Millman, Brett, Barnowski, & Poline, 2018). Successful application of replication analysis principles has provided key advances in the neuroimaging of speech perception (Evans, 2017). We attempted to apply the same principles of throughout our analysis by performing a large scale, powered replication analysis of the key finding, in full communication of methods and availability of data used.

One limitation of this study is that our analysis only included healthy individuals (i.e., those who are assumed not to have sub‐clinical or clinical brain damage), limiting the generalisation of our findings to clinical cohorts. There is a paucity of available high‐quality imaging data in this vulnerable population. A second limitation is that our Replication Cohort did not closely match the Discovery Cohort in terms of age, with a difference in medians of 10 years; they also used different tests of emotional function. This was due to the limited availability of datasets which have both multi‐shell diffusion data and detailed neurocognitive testing. While the regression model in the Replication Cohort was still significant, the magnitude of the regression slope was smaller than that observed in the Discovery Cohort. However, this is not unexpected: in psychology, effect sizes reported in replication studies are generally half of that reported in original studies (Open Science Collaboration, 2015; van Aert & van Assen, 2018). Further, the significant replication of results, despite the age mismatch between the Discovery and Replication cohorts, may suggest that the observed effects are robust to differences in age. Our approach using a specific, pre‐defined composite cluster was not subject to the “model degrees of freedom” which purportedly have driven false‐positive replications in neuroimaging (Hong, Yoo, Han, Wager, & Woo, 2019). Last, our study did not make use of multivariate data mining or machine learning approaches which are gaining popularity with application in prediction studies, and show high levels of accuracy (Benedict et al., 2004; Dyrba et al., 2015; Moradi, Pepe, Gaser, Huttunen, & Tohka, 2015).

## 5 CONCLUSION

We identified structural brain signatures of white matter structure which are sensitive to and specific for cognitive function. The tracts involved are both anatomically‐precise and correspond to functional hubs derived from a meta‐analysis of all available functional neuroimaging literature. The association of our emotional performance signature was replicated in a large independently acquired cohort. These results provide convergent evidence of a significant structural contribution to emotional and cognitive performance, even in normal healthy people. While our findings raise the possibility that MRI may, with further testing, be able to provide objective and quantifiable indicators of subclinical brain changes (e.g., in psychiatric illness, concussion or early dementia), there is a long way to go in bringing dMRI to clinical utility. Developing such markers could be a useful first step toward transforming dMRI into a clinical tool to track brain changes, relating directly to function, in a way that matters to patients.

## CONFLICT OF INTEREST

The authors report no conflicts of interest.

## Supporting information


**Appendix S1**: Supplementary Material

## Data Availability

The HCP Young Adult Cohort data are available at https://www.humanconnectome.org. Our analysis scripts are available at https://github.com/t-welton/structural_biomarker. Statistical maps from TBSS and VBM analyses were uploaded to https://neurovault.org/.
