# Supplementary material for: Replicable brain signatures of emotional bias and memory based on diffusion kurtosis imaging of white matter tracts
Source: Hum Brain Mapp. 2019 Nov 26;41(5):1274–85. doi: 10.1002/hbm.24874 (PMC7268065; doi:10.1002/hbm.24874)
Supplement: Supplementary file 1 — Appendix S1: Supplementary Material [file HBM-41-1274-s001.docx]

**Supplemental Information**

1. Method for construction of the sham signature.

Using the data described in Supplemental Information 6, we defined the cortico-spinal tract by, first, placing a 5-mm seed sphere in the white matter adjacent to the motor cortex in the precentral gyrus and a target region placed in the brainstem at the second-most inferior slice. We generated 1,000,000 tracks using probabilistic tractography and converted the result into a track density image {Calamante, 2010 #53}. We applied an incrementally increasing threshold to the track density image until the resulting binary mask was narrow, centred in the cortico-spinal tract. Using nonlinear registrations created in FNIRT ([Andersson, Jenkinson et al. 2007](#_ENREF_2)), the corticospinal tract masks were registered to the space of the individual subjects.

2. One-sample t-tests for equality with 0 for age- gender- and education-corrected cognitive data. Normalisation was performed against a large sample (n>10,000) of healthy individuals from the Brain Resource International Database ([Silverstein, Berten et al. 2007](#_ENREF_7)). No cognitive measure was significantly different from 0, thus, our cohort was considered normal for all cognitive measures.

| **Cognitive Measure** | **t** | **p** |
| --- | --- | --- |
| **Negativity Bias** | 0.144 | 0.886 |
| **Emotional Resilience** | 0.034 | 0.973 |
| **Social Skills** | 0.398 | 0.691 |
| **Depressed Mood** | 0.065 | 0.949 |
| **Anxiety** | 0.341 | 0.733 |
| **Stress** | 0.283 | 0.777 |
| **Motor Tapping** | 0.225 | 0.822 |
| **Impulsivity** | -0.400 | 0.690 |
| **Attention** | -0.572 | 0.568 |
| **Information Processing** | -1.491 | 0.138 |
| **Memory** | 0.798 | 0.426 |
| **Executive** | -0.874 | 0.384 |
| **Verbal Interference** | -0.990 | 0.324 |
| **Emotional Identification** | -1.345 | 0.180 |
| **Emotional Bias** | -1.665 | 0.098 |

3. Rotated component matrix showing the loadings from the principal components analysis. Loadings lower than 0.2 are supressed for clarity.

|  | **Negative Affect** | **Emotion** | **Executive** | **Memory** | **Verbal** |
| --- | --- | --- | --- | --- | --- |
| **Negativity Bias** | 0.934 |  |  |  |  |
| **Emotional Resilience** | -0.324 |  |  | 0.891 |  |
| **Social Skills** | -0.235 |  |  |  |  |
| **Depressed Mood** | 0.881 |  |  |  |  |
| **Anxiety** | 0.869 |  |  |  |  |
| **Stress** | 0.913 |  |  |  |  |
| **Motor Tapping** |  |  |  |  |  |
| **Impulsivity** |  |  |  |  |  |
| **Attention** |  |  |  |  |  |
| **Information Processing** |  |  | 0.738 |  | 0.229 |
| **Memory** |  |  | -0.672 | 0.397 | -0.251 |
| **Executive** |  |  | 0.744 |  |  |
| **Verbal Interference** |  |  |  |  | 0.952 |
| **Emotional Identification** |  | 0.880 |  |  |  |
| **Emotional Bias** |  | 0.899 |  |  |  |

4. Non-significant trends in t-statistic maps for the tract-based spatial statistics analysis (t>2.5).


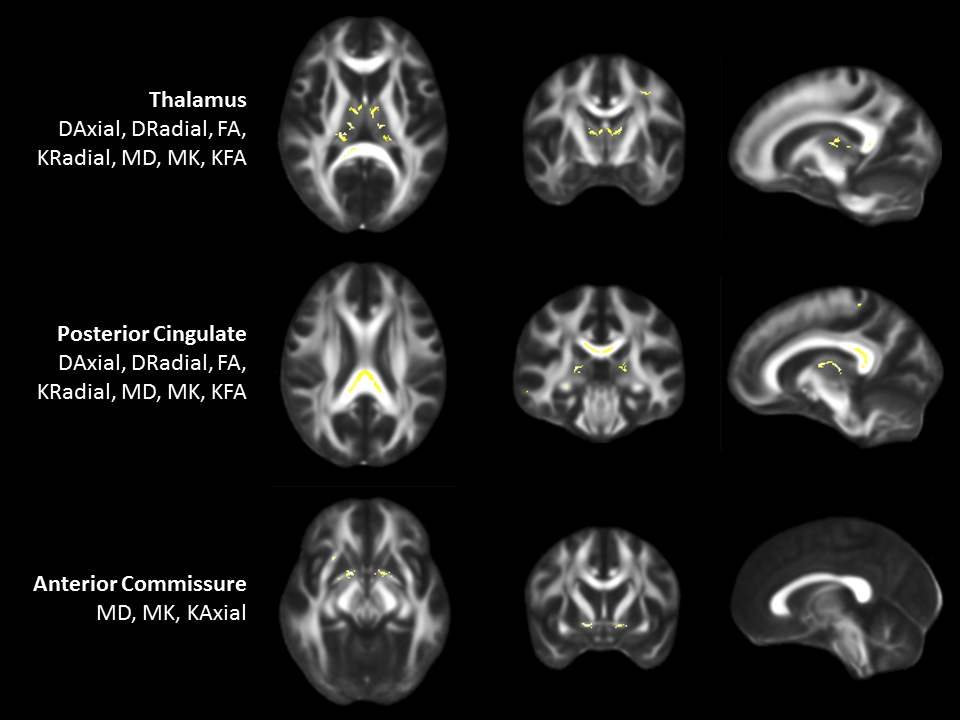


5. A significant cluster identified in the voxelwise test across all intracranial voxels for covariance with the five cognitive and emotional components.


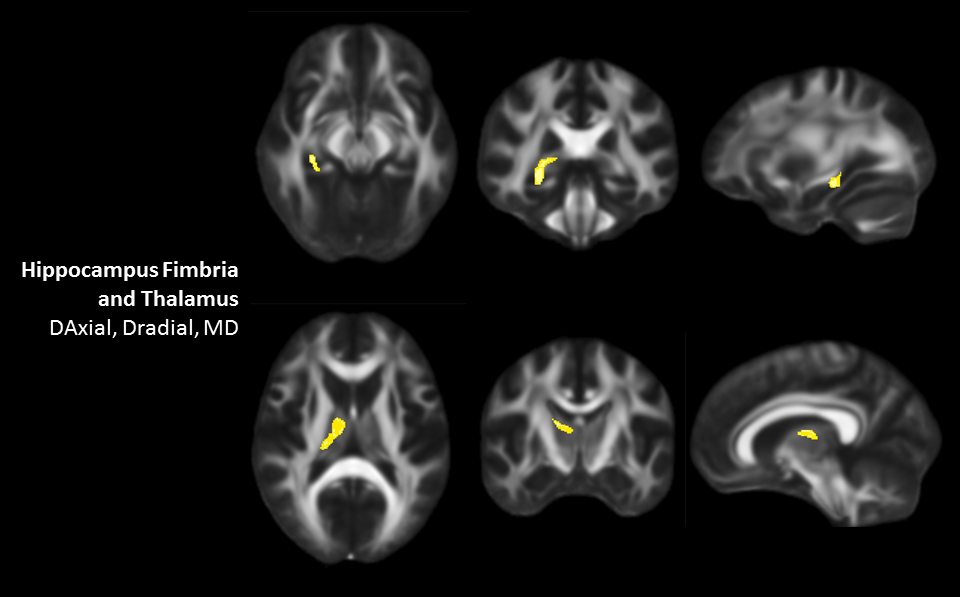


6. Methods for the probabilistic diffusion tractography for visualisation of the identified tracts.

One healthy individual underwent MRI on a GE Compact 3T scanner (peak gradient amplitude 80 mT/m, slew rate 700 T/m/s) ([Lee, Mathieu et al. 2016](#_ENREF_6), [Weavers, Shu et al. 2016](#_ENREF_10), [Foo, Laskaris et al. 2018](#_ENREF_3)) under an IRB-approved protocol. To account for additional concomitant fields arising from the asymmetric transverse gradients, frequency shifting ([Weavers, Tao et al. 2018](#_ENREF_11)) and gradient pre-emphasis ([Tao, Weavers et al. 2017](#_ENREF_9)) were applied. High-order gradient non-linearity correction with even-order terms was applied ([Tao, Trzasko et al. 2017](#_ENREF_8)). The low-distortion MUSE ([Chen, Guidon et al. 2013](#_ENREF_2)) diffusion sequence was applied: 1.2 mm^3^, TE=54.5 ms, TR=12500 ms, FA=90°, 33 diffusion-weighted volumes at b=1000 mm/s^2^ and 1 b=0 volume, in-plane acceleration factor=2, ~15 minutes scan time.

The dataset was denoised, corrected for susceptibility, eddy-currents and motion using TOPUP and eddy_cuda ([Andersson and Sotiropoulos 2016](#_ENREF_1)). A white-matter response function was generated using the Dhollander method and fibre orientation distributions created using constrained spherical deconvolution ([Jeurissen, Tournier et al. 2014](#_ENREF_5)). Probabilistic tractography with 100,000 tracks was performed using the significant cluster as a seed region after it was transformed from MNI space to the single-subject diffusion space using affine registration ([Jenkinson, Bannister et al. 2002](#_ENREF_4)).

7. Non-significant trends in t-statistic maps for the voxel-based morphometry analysis (t>2.5). A single main cluster was identified in the hippocampus which was associated with larger volumes of grey matter (red-yellow) overlaid on the average grey matter image.


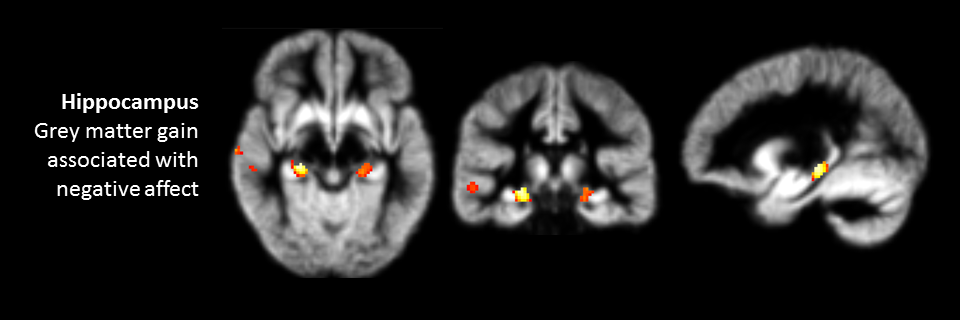


8. Scatter plots of the structural brain signatures’ relationship to emotional bias and emotional memory.


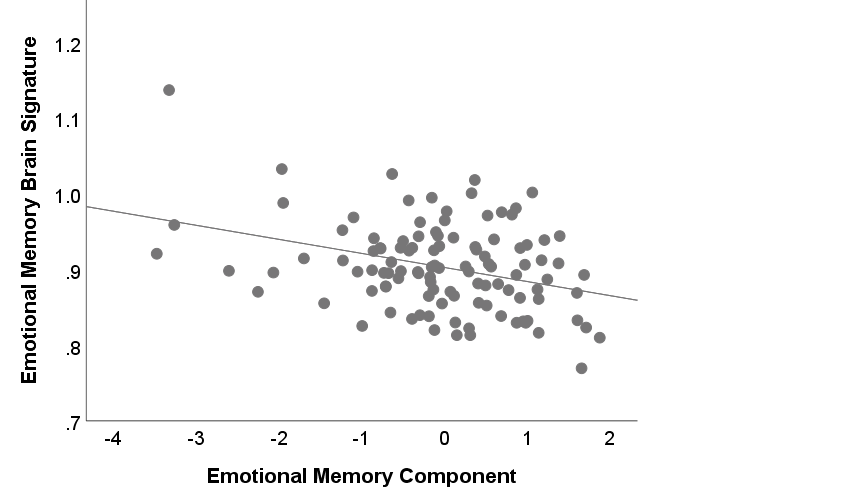


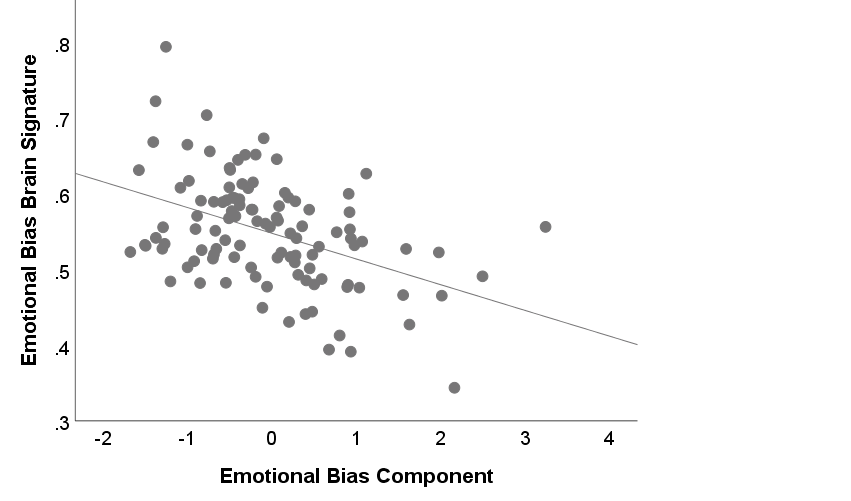


9. Results of regression analyses of the emotional bias structural biomarker and negative affect, executive function, memory and verbal principal components.

| **Independent Variable (block 1, enter)** | **Age, Gender, Education (block 2, stepwise)** | **R^2^** | **F(1,172)** | **p** |
| --- | --- | --- | --- | --- |
| **Negative Affect** | Below threshold for inclusion | 0.012 | 1.27 | 0.26 |
| **Executive** | Below threshold for inclusion | 0.014 | 1.56 | 0.21 |
| **Memory** | Below threshold for inclusion | -0.000 | 0.01 | 0.92 |
| **Verbal** | Below threshold for inclusion | -0.008 | 0.10 | 0.75 |

10. Results of regression analyses of the emotional memory structural biomarker and negative affect, executive function, emotional bias and verbal principal components.

| **Independent Variable (block 1, enter)** | **Age, Gender, Education (block 2, stepwise)** | **R^2^** | **F(1,172)** | **p** |
| --- | --- | --- | --- | --- |
| **Negative Affect** | Below threshold for inclusion | 0.013 | 1.47 | 0.23 |
| **Executive** | Below threshold for inclusion | 0.014 | 1.54 | 0.22 |
| **Emotional Bias** | Below threshold for inclusion | 0.010 | 1.07 | 0.30 |
| **Verbal** | Below threshold for inclusion | 0.023 | 2.59 | 0.11 |

**References**

Andersson, J. L. R. and S. N. Sotiropoulos (2016). "An integrated approach to correction for off-resonance effects and subject movement in diffusion MR imaging." Neuroimage **125**: 1063-1078.

Chen, N. K., A. Guidon, H. C. Chang and A. W. Song (2013). "A robust multi-shot scan strategy for high-resolution diffusion weighted MRI enabled by multiplexed sensitivity-encoding (MUSE)." Neuroimage **72**: 41-47.

Foo, T. K. F., E. Laskaris, M. Vermilyea, M. Xu, P. Thompson, G. Conte, C. Van Epps, C. Immer, S.-K. Lee, E. T. Tan, D. Graziani, J.-B. Mathieu, C. J. Hardy, J. F. Schenck, E. Fiveland, W. Stautner, J. Ricci, J. Piel, K. Park, Y. Hua, Y. Bai, A. Kagan, D. Stanley, P. T. Weavers, E. Gray, Y. Shu, M. A. Frick, N. G. Campeau, J. Trzasko, J. Huston III and M. A. Bernstein (2018). "Lightweight, compact, and high-performance 3T MR system for imaging the brain and extremities." Magnetic Resonance in Medicine **80**(5): 2232-2245.

Jenkinson, M., P. Bannister, M. Brady and S. Smith (2002). "Improved optimization for the robust and accurate linear registration and motion correction of brain images." Neuroimage **17**(2): 825-841.

Jeurissen, B., J. D. Tournier, T. Dhollander, A. Connelly and J. Sijbers (2014). "Multi-tissue constrained spherical deconvolution for improved analysis of multi-shell diffusion MRI data." Neuroimage **103**: 411-426.

Lee, S. K., J. B. Mathieu, D. Graziani, J. Piel, E. Budesheim, E. Fiveland, C. J. Hardy, E. T. Tan, B. Amm, T. K. Foo, M. A. Bernstein, J. Huston, 3rd, Y. Shu and J. F. Schenck (2016). "Peripheral nerve stimulation characteristics of an asymmetric head-only gradient coil compatible with a high-channel-count receiver array." Magn Reson Med **76**(6): 1939-1950.

Silverstein, S. M., S. Berten, P. Olson, R. Paul, L. M. Willams, N. Cooper and E. Gordon (2007). "Development and validation of a World-Wide-Web-based neurocognitive assessment battery: WebNeuro." Behav Res Methods **39**(4): 940-949.

Tao, S., J. D. Trzasko, J. L. Gunter, P. T. Weavers, Y. Shu, J. Huston, S. K. Lee, E. T. Tan and M. A. Bernstein (2017). "Gradient nonlinearity calibration and correction for a compact, asymmetric magnetic resonance imaging gradient system." Phys Med Biol **62**(2): N18-n31.

Tao, S., P. T. Weavers, J. D. Trzasko, Y. Shu, J. Huston, 3rd, S.-K. Lee, L. M. Frigo and M. A. Bernstein (2017). "Gradient pre-emphasis to counteract first-order concomitant fields on asymmetric MRI gradient systems." Magnetic resonance in medicine **77**(6): 2250-2262.

Weavers, P. T., Y. Shu, S. Tao, J. Huston, 3rd, S. K. Lee, D. Graziani, J. B. Mathieu, J. D. Trzasko, T. K. Foo and M. A. Bernstein (2016). "Technical Note: Compact three-tesla magnetic resonance imager with high-performance gradients passes ACR image quality and acoustic noise tests." Med Phys **43**(3): 1259-1264.

Weavers, P. T., S. Tao, J. D. Trzasko, L. M. Frigo, Y. Shu, M. A. Frick, S.-K. Lee, T. K. F. Foo and M. A. Bernstein (2018). "B(0) concomitant field compensation for MRI systems employing asymmetric transverse gradient coils." Magnetic resonance in medicine **79**(3): 1538-1544.
